# Protamines and the sperm nuclear basic proteins Pandora’s Box of insects

**DOI:** 10.1139/bcb-2023-0363

**Published:** 2024-02-26

**Authors:** Melissa R. Leyden, Brent Gowen, Rodrigo Gonzalez-Romero, Jose Maria Eirin-Lopez, Bo-Hyun Kim, Fumio Hayashi, Jay McCartney, Patrick C. Zhang, Miyoko Kubo-Irie, Jeffrey Shabanowitz, Donald F. Hunt, Patrick Ferree, Harold Kasinsky, Juan Ausió

**Affiliations:** a Department of Chemistry, University of Virginia, Charlottesville, VA 22904, USA; b Department of Biology, University of Victoria, Victoria, BC V8W 2Y2, Canada; c Department of Biochemistry and Microbiology, University of Victoria, Victoria, BC V8W 2Y2, Canada; d Environmental Epigenetics Laboratory, Institute of Environment, Florida International University, Miami, FL, USA; e Florida International University, Miami, FL, USA; f Department of Biology, Tokyo Metropolitan University, Minamiosawa 1-1, Hachioji, Tokyo 192-0397, Japan; g Institute of Natural Sciences, Massey University, Palmerston North, Manawatu, New Zealand; h W.M. Keck Science Department, Claremont McKenna, Pitzer, and Scripps Colleges, Claremont, CA 91711, USA; i Biological Laboratory, The Open University of Japan, Wakaba, Mihama-ku, Chiba, 261-8506, Japan; j Department of Pathology, University of Virginia, Charlottesville, VA 22903, USA; k Department of Zoology, University of British Columbia, Vancouver, BC V6T 1Z4, Canada

**Keywords:** sperm nuclear basic proteins (SNBPs), protamines, insects, mass spectrometry/Edman N-terminal sequencing

## Abstract

Insects are the largest group of animals when it comes to the number and diversity of species. Yet, with the exception of *Drosophila*, no information is currently available on the primary structure of their sperm nuclear basic proteins (SNBPs). This paper represents the first attempt in this regard and provides information about six species of Neoptera: *Poecillimon thessalicus*, *Graptosaltria nigrofuscata*, *Apis mellifera*, *Nasonia vitripennis*, *Parachauliodes continentalis*, and *Tribolium castaneum*. The SNBPs of these species were characterized by acetic acid urea gel electrophoresis (AU-PAGE) and high-performance liquid chromatography fractionated. Protein sequencing was obtained using a combination of mass spectrometry sequencing, Edman N-terminal degradation sequencing and genome mining. While the SNBPs of several of these species exhibit a canonical arginine-rich protamine nature, a few of them exhibit a protamine-like composition. They appear to be the products of extensive cleavage processing from a precursor protein which are sometimes further processed by other post-translational modifications that are likely involved in the chromatin transitions observed during spermiogenesis in these organisms.

## Graphical Abstract

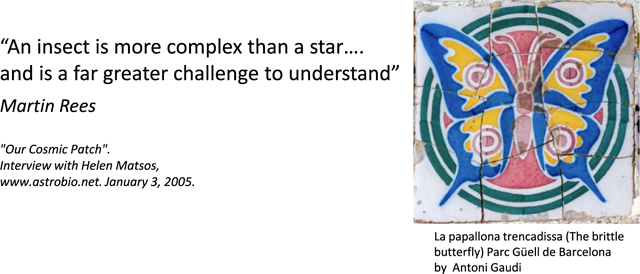

## Introduction

During eukaryote sexual reproduction, two sets of haploid male (sperm) and female (egg) gametes, arising from a unique meiosis cell division event combine during fertilization to produce a diploid zygote. The size of the gametes may be similar (isogamy) or quite different (anysogamy) with a large number of forms having a very small sperm (oogamy) (Kirk 2006). The oogamus sperm is characterized by the presence of a small nucleus with highly compacted chromatin in which a large amount (>90%) of the genome is transcriptionally silent. To achieve this, DNA is associated with a variety of sperm-specific chromosomal proteins referred to as sperm nuclear basic proteins (SNBPs) (Ausió 1995; Eirin-Lopez and Ausio 2009; Saperas and Ausio 2013). Three main types of SNBP have been described: the histone (H), protamine like (PL), and protamine type (P) (Ausió 1999). Although quite an extensive characterization of these groups has been carried out over the past 40 years, both in invertebrate (Kasinsky 1989; Rocchini et al. 1995; Ausió et al. 1997; Lewis et al. 2003; Torok et al. 2023) and vertebrate (Ausio et al. 2007; Eirin-Lopez and Ausio 2009; Saperas and Ausio 2013) animals and, to a much lesser extent, in plants (Borg and Berger 2015), very little, if any, is known about the SNBPs of insects (Kasinsky 1989).

With an estimate of 5.5–7.0 million insects and terrestrial arthropod species (Stork 2018), this group of organisms represents the most diverse of all animal groups (van Klink et al. 2022). Yet, with the exception of the *Drosophila melanogaster* protamine (Jayaramaiah Raja and Renkawitz-Pohl 2005), no other SNBP amino acid sequence information is available for this group. Early attempts in this direction (summarized in (Bloch 1969, 1976) and in (Kasinsky 1989)) produced qualitative data mainly based on the staining characteristics of chromatin in the sperm nuclei which hinted to the presence of cysteine in some species analyzed, and provided an early glimpse to the potential protein diversity involved. A recent functionally related example of this is provided by the manifold organization and transitions undergone by chromatin during spermiogenesis of these organisms (Kasinsky et al. 2021). Indeed, all this molecular and cytological evidence mirrors the diverse characteristics of the different species of insect and it does not come as a surprise. Insects have the ability to evolve very rapidly in response to global environmental changes (Garnas 2018) and so do the associated molecular traits (McCulloch and Waters 2023). For instance, it has been shown that rapid genomic evolution drives the diversification of male reproductive genes in dung beetles (Mrinalini et al. 2021). Part of this success relies on their ability to produce one or more phenotypes from one single genotype (Polyphenism) that allows them to adapt to those changes (Simpson et al. 2011).

This paper represents a brief summary of work in insect SNBPs that started in our labs (HK and JA) in the 1990’s that has involved the collection of significantly large amounts of sperm gathered from species provided to us by several groups around the world during these years. Part of this slow and lengthy progress can be attributed in part, like in the case of the hiatus on histone/protamine research from the 1870s–1890s to the 1950s (Ando et al. 1973; van Holde 1988), to the lack of suitable analytical technologies. Although of a different nature, a similar lack of techniques to analyze, in this case, the minute amounts of SNBPs from the small amounts of sperm obtained from insects hindered the steady pace of the work. The high abundance of arginine in canonical protamines (Balhorn 2007; Kasinsky et al. 2012), similar to ribosomal proteins, additionally complicated the issue. Only with the advent of powerful techniques such as intact protein sequencing by liquid chromatography–nano–electrospray ionization–mass spectrometry (LCMS) (Coon et al. 2005) has it been ultimately possible to overcome such difficulties (D’Ippolito et al. 2019).

Despite the technological advances mentioned above, the topic remains challenging. For instance, regardless of the current availability of genomic information for an increasing number of insect species (Li et al. 2019), the use of bioinformatics tools to search for a protamine/protamine-like sequence is extremely arduous and often unsuccessful. This is mainly because of the intrinsic complexity and the expected diversity of SNBPs in this group of animals which hampers their proper annotation. To this endeavor, the quote at the opening of this section by Martin Rees remains very fitting. It is our hope that some of the limited information provided here will incentivize future research into the field.

## Materials and methods

### SNBP extraction

Two different extraction methods were used. For SNBPs lacking cysteine, starting sperm-containing material (spermatheca, seminal vesicles) were re-suspended in 0.15 mol/L NaCl, 10 mmol/L Tris–HCl (pH 7.5), 0.5% Triton X-100 in the presence of 1:100 Complete Protease Cocktail Inhibitor (Roche, Basel, Switzerland) and homogenized with a Dounce homogenizer. A ratio of approximately 2 volumes of buffer per volume of starting material was used. The suspension was then incubated on ice for 10 min and the sample was centrifuged at 3000 *g* for 10 min at 4 °C. The nuclear pellet was re-suspended in the same buffer without detergent and was centrifuged again under the same conditions. The pellets thus obtained were then homogenized using a Dounce homogenizer (10 strokes) in approximately 6–8 volumes of 0.6 N HCl. The HCl suspension obtained in this way was centrifuged at 16 000 × *g* for 10 min at 4 °C and the clear supernatant was precipitated with 6 volumes of acetone at −20 °C overnight.

To extract cysteine-containing SNBPs, cysteine residues were reduced and alkylated by pyridylethylation previous to the HCl extraction (McKay et al. 1986). To this end, approximately 6 volumes of 4 mol/L guanidinium chloride, 50 mmol/L Tris–HCl (pH 7.5), 1.25 mmol/L EDTA buffer with 1:100 Complete Protease Cocktail Inhibitor, were added to the starting sample followed by Dounce homogenization. The homogenate was incubated for 10 min at 4 °C and ß-mercaptoethanol was added to a final concentration of 50 mmol/L, and incubated for an additional 90 min at room temperature in the dark (covering the tubes with aluminum foil). Two microliters of 2-vinylpyridine were added for every 250 μL of solution and incubated for an additional 30 min in the dark with vortexing every 5 min. At that point, the sample was diluted 1:10 *v*:*v* with distilled water (to bring the guanidinium concentration to about 0.4 mol/L or less and centrifuged at 16 000 × *g* for 10 min at 4 °C. The pellet thus obtained was re-suspended in 0.6 N HCl (100 μL for every 250 μL of the initial starting suspension) and homogenized with a Dounce homogenizer. The suspension was centrifuged at 16 000 × *g* for 10 min at 4 °C and the clear supernatant was precipitated with 6 volumes of acetone at −20 °C overnight. The final acetone precipitates at the end of each extracting procedure were centrifuged at 16 000 × *g* for 10 min at 4 °C and the pellets were dried using a speed vac device. Samples to be analyzed by AU-PAGE were dissolved in (4 mol/L urea, 5% acetic acid) sample buffer.

### Gel electrophoresis

Acetic acid (5%)–urea (2.5 mol/L) polyacrylamide gel electrophoresis was carried out according to Hurley (Hurley 1977), as described elsewhere (Ausió 1992).

### Reversed phase HPLC (rpHPLC)

High-performance liquid chromatography (HPLC) was performed as described in (Ausió and Moore 1998; Cheema and Ausio 2017). In brief, a 100 μL aliquot of the protein extracts were injected onto a C_18_ column (Vydac, Hesperia, CA, USA) (4.6 × 250 mm, particle size: 5 μm, pore size: 300 Å) and eluted at 1 mL/min using a mobile phase consisting of (0.1% trifluoroacetic acid) and acetonitrile gradient. Samples were fractionated on a Beckman Coulter SYSTEM GOLD^®^ 126 Solvent Module equipped with SYSTEM GOLD^®^ 168 Detector.

### Transmission electron microscopy (TEM)

#### Figures 2F and 4D (Ferree et al. 2019)

All individuals were decapitated and the testes isolated and fixed in 3% formaldehyde and 3% glutaraldehyde in 0.1 mol/L sodium cacodylate buffer. After buffer and 50% ethanol washes, the testes were in bloc stained for 1 h in 1% uranyl acetate in 50% ethanol. The testes were then dehydrated through a graded ethanol series and embedded into EMbed 812 resin (Epon), using propylene oxide as a transition fluid. The EMbed was polymerized at 60 °C for 48 h. TEM sections were contrasted with uranyl acetate and lead citrate and viewed in a Hitachi H7000 TEM operating at 75 kV. Images were captured using an AMT (Advanced Microscopy Techniques, Woburn, MA, USA) 2k × 2k CCD camera. All chemicals were supplied by EMS (Electron Microscopy Sciences, Hatfield, PA, USA).

#### Figure 8D (Dias et al. 2015)

The testes and seminal vesicles of *Tribolium corretto* were dissected in 0.1 mol/L sodium cacodylate buffer, pH 7.2, and fixed in a 2.5% glutaraldehyde solution containing 0.2% picric acid, 3% sucrose, and 5 mmol/L CaCl2 in the above buffer, for approximately 24 h. The material was post-fixed in a 1% osmium tetroxide solution for 2 h, dehydrated in an increasing alcohol series, infiltrated and finally embedded in epoxy resin (Epon 812). Ultrathin sections obtained with a Reichert Ultracut II E ultramicrotome were routinely stained and then observed with a Philips CM 10 electron microscope operating at 80 kV.

### Phylogenetic analysis

#### Sequence alignment

The protamine of *Trilobium castaneum* was retrieved through recurrent BLAST searches of its genome on GenBank databases. The complete sequences were edited and aligned to those of the bee protamine based on their amino acid sequences, using the BIOEDIT (Hall 1999) and CLUSTAL_X programs using the default parameters as described elsewhere (Ishibashi et al. 2009).

##### Estimation of evolutionary rates

The rate of evolution of the bee protamines evolution was estimated by calculating the number of amino acid substitutions per site and plotted against the evolutionary distance between the bee species analyzed. Divergence times between taxa were defined according to Hedges et al. (2006).

#### Edman degradation protein sequencing

Peptide sequencing was performed on an AB1 Model 470A gas-phase protein sequenator. The standard AB1 02C Ser program was used for coupling and cleavage with the cartridge set at 40 °C. Phenylthiohydantoin derivatives were obtained with trifluoroacetic acid at 55 °C. These derivatives were analyzed on a Beckman Microflow HPLC (Downing and Mann 1976) system equipped with an IBM cyano column, 2–3 nmol of protein/peptide were used in the analysis.

### Mass spectrometry

#### Materials

Pierce LCMS-grade water and formic acid were purchased from Thermo Scientific (Rockford, IL). LCMS-grade acetonitrile and 2-propanol were purchased from Honeywell (Charlotte, NC). Acetic acid was purchased from Sigma Aldrich (St. Louis, MO).

##### Liquid chromatography–nano-electrospray ionization–tandem mass spectrometry (LCMS)

For hydrophilic interaction chromatography (HILIC) 1 μL of extracted protein solution was diluted 10-fold with 0.1% acetic acid in acetonitrile. Approximately 1 μL (10%) of the dilution, corresponding to ~0.15% of the total suspension of all samples, was pressure-loaded onto in-house prepared pre-columns (360 μm OD × 100 μm ID) (Udeshi et al. 2008). Both analytical and pre-columns had a 2 mm Kasil 1624 frit and the analytical column (360 μm OD × 75 μm ID) integrated with an electrospray tip (Ficarro et al. 2009). The HILIC pre-column was packed to 7 cm with 12 μm diameter, 300 Å PolyHYDROXYETHYL A (PHEA) packing material from PolyLC Inc. (Columbia, MD), and was connected to an analytical column packed to 10 cm with 5 μm diameter, 300 Å PHEA packing material. For reverse-phase chromatography 10% of the extracted protein solution was pressure loaded onto a reverse-phase column in 0.1% acetic acid. The reverse-phase pre-column was packed to 7 cm with 10 μm diameter, 300 Å PLRP-S packing material from Agilent (Santa Clara, CA) and connected to an analytical column packed to 10 cm with 3 μm diameter, 300 Å PLRP-S packing material.

An Agilent Technologies (Santa Clara, CA) 1100 Series Binary HPLC system coupled to a Thermo Scientific Orbitrap Fusion Tribrid mass spectrometer (San Jose, CA) operated in low pressure intact protein mode was used to analyze the proteins in each sample.

The PLRP-S pre-column was rinsed with 100% solvent A (0.3% formic acid in water) for 20 min at a flow rate of ~3 μL/min then connected to the PLRP-S analytical column. Proteins were eluted using a gradient of 0%–60%–100% solvent B (72% acetonitrile, 18% 2-propanol, 10% water, and 0.3% formic acid) in 0–60–70 min at a flow of ~100 nL/min. Highly basic proteins are not retained well on reverse-phase columns (Buszewski and Noga 2012). For this reason, HILIC was used to retain highly hydrophilic proteins in the samples. The PHEA-packed pre-column was washed with solvent B (95% acetonitrile, 15% water, and 0.2% acetic acid) for 20 min at a flow rate of ~3μL/min then connected to a PHEA-packed analytical column. Proteins were eluted using a gradient of 100%–0% solvent B for 60 min with a 10 min hold of 100% solvent A (0.5% acetic acid in water) before reequilibrating the column back to 100% solvent B at a flow rate of ~100 nL/min.

Proteins were selected for fragmentation from a 60 000 resolution Orbitrap MS1 scan. Using a 3 s cycle time, proteins with a charge state of ≥3 were isolated by the quadrupole with an isolation window of 2 m/z and fragmented electron transfer dissociation (ETD) and collisional dissociation (Syka et al. 2004). MS2 scans were acquired in the Orbitrap at 60 000 or 120 000 resolution with an automatic gain control target of 1e5.

#### LCMS data analysis

MS1 and MS2 spectra were manually inspected using Qual Browser (Thermo Scientific). MS2 ETD spectra were deconvolved using the Xtract algorithm (Thermo Scientific) (Senko et al. 1995). The protein sequences were determined by manual de novo analysis of MS2 spectra.

##### Matrix-assisted laser desorption ionization (MALDI)

These types of analyses were carried out as described previously (Hunt et al. 1991, 1996; Carlos et al. 1993b). The monoisotopic and average masses (MH^+^) from the protein sequences were calculated using Mac ProMass Program Version 1.05.

## Results

### The insect players

**Fig. 1.**
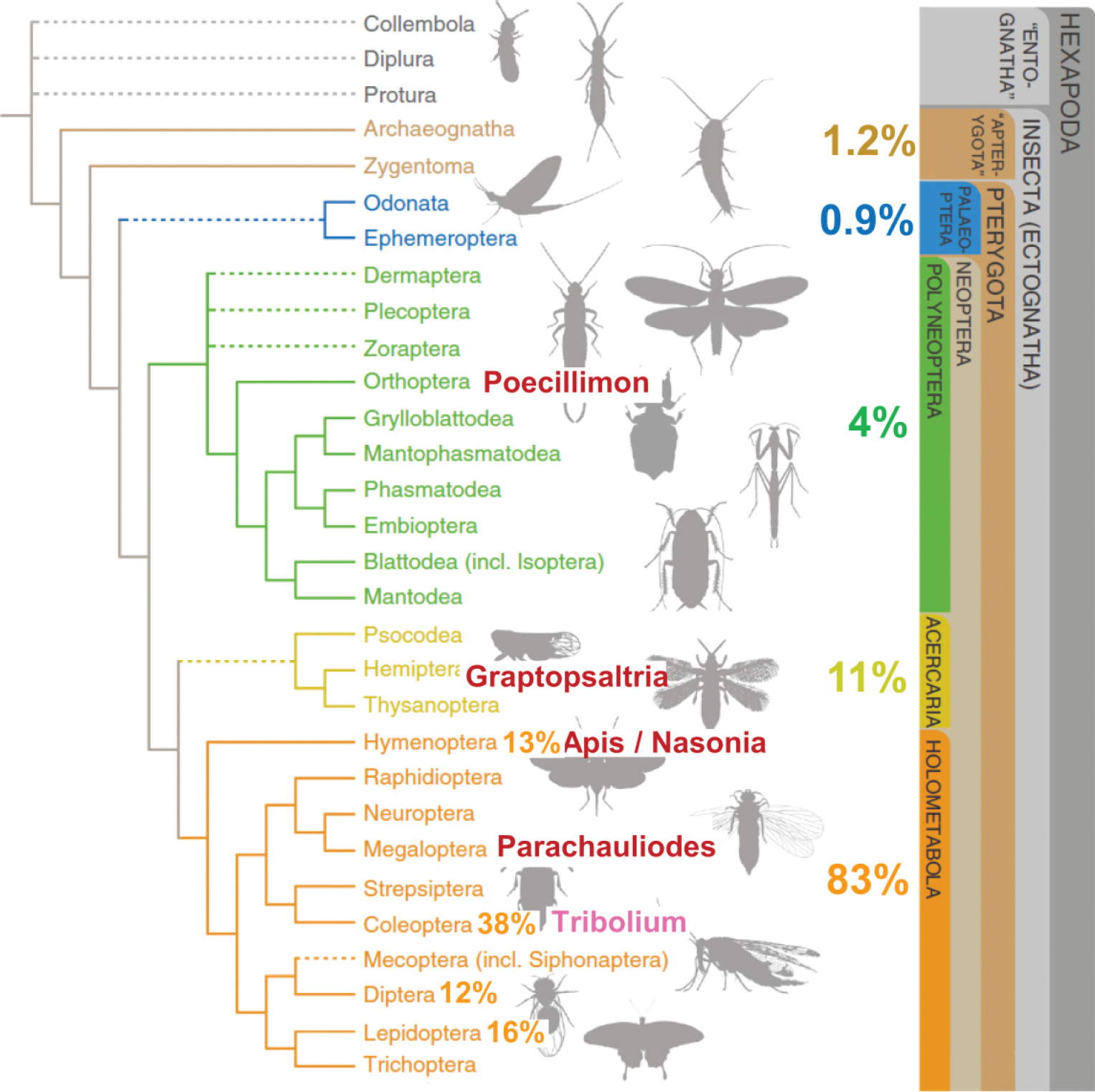
Insect phylogeny (Tihelka et al. 2021) with the names of the species used in this work and for which the sperm nuclear basic proteins were determined experimentally (red) or obtained from data mining of existing genomes (pink). The number of species belonging to several of the different lineages and orders (Engel 2015) is also indicated. Figures modified from (Tihelka et al. 2021) and reproduced with permission from Elsevier.

Figure 1 shows a phylogenetic tree of insects (Tihelka et al. 2021) with the percentile of insect species corresponding to the different Superorders (Engel 2015). The names of the insects whose SNBPs have been characterized in this work are indicated: *Poecillimon thessalicus* (Brunner von Wattenwyl, 1891); *Graptosaltria nigrofuscata* (Motschulsky, 1866); *Apis mellifera* (Linnaeus, 1758); *Nasonia vitripennis* (Walker, 1836); *Parachauliodes continentalis* (Van der Weele, 1909); and *T. castaneum* (Herbst, 1797). These species were collected in Eurasia and North America.

### Setting the stage with the bee protamines

**Fig. 2.**
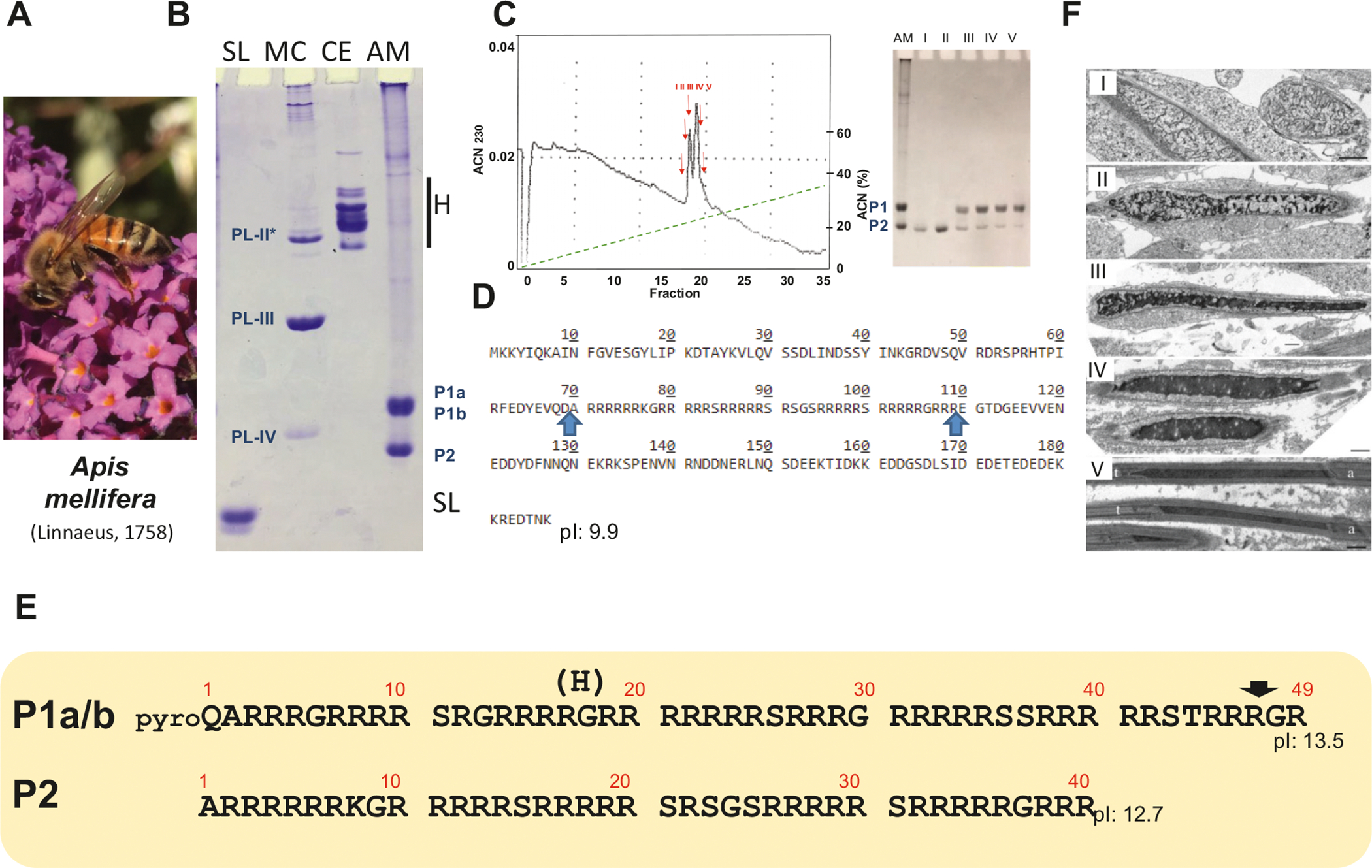
Spermiogenesis and protamine of the honeybee. (A) The honeybee *Apis mellifera*. (B) AU-PAGE of the sperm nuclear basic proteins (SNBPs) of *A. mellifera* (AM) sperm in comparison to the SNBPs from salmon *Oncorhynchus keta* (salmine protamine, SL), California mussel (*Mytilus californianus*, MC), and somatic histones from chicken erythrocytes (CE). The PL-II*, PL-III, and PL-IV from *M. californianus* (Carlos et al. 1993a) are indicated. (C) Reversed phase HPLC analysis of the HCl-extracted SNBPs from sperm of *A. mellifera*. An AU-PAGE of the fractions (I–V) collected is also shown. (D) Protein sequence of the protamine P2 precursor identified with a BLAST search of the BeeBase (https://hymenoptera.elsiklab.missouri.edu/beebase) against the experimentally determined (N-terminal Edman degradation sequencing) protein sequence of *A. mellifera* P1. (E) Protamine sequence of P1a, P1b, and P2 using Edman N-terminal degradation sequencing and mass spectrometry analysis. The black arrow represents an LCMS identified cleavage site. (F) Electron micrographs at different stages of spermiogenesis. I = longitudinally and cross-sectioned nuclei from early spermatids in which chromatin fibers start to coalesce. Chromatin fiber thickening (II–IV) progresses in late spermatids until the sperm are mature (V) with electron dense nuclei. At this stage, microtubules longitudinally aligned above and below the sectioned nuclei can be observed. The acrosomes (a) and tails (t) are indicated within the image. Scale bars = 500 nm.

The first insect analyzed in this project was the honey bee *A. mellifera*. The amount of protein material gathered from drone semen of this insect (Fig. 2B) was large enough to allow for an rp-HPLC fractionation (Fig. 2C) and, at the time (1998), it was suitable for N-terminal Edman degradation sequencing. As shown in Fig. 2B, three main electrophoretic bands (P1a-b and P2) can be clearly visualized with an electrophoretic mobility centered around that of the lysine-rich PL-IV from the California mussel (*Mytilus californianus*) which has 60 amino acids and a molecular mass of 6450 Da (Carlos et al. 1993a). Edman degradation of fraction II (Fig. 2C) produced the sequence of 40 amino acids comprised between the two arrows in Fig. 2D with a molecular mass of 5509.4 Da which was in good agreement with the mass of 5508.9 determined by MALDI for this fraction.

The amino acid composition analyses carried out with fractions I–V (Fig. 2C) exhibited an almost identical arginine rich composition. However, attempts to obtain the amino acid sequence for protein P1 and fractions III–V (Fig. 2C) using the Edman degradation approach turned out to be unsuccessful at the time, and the nature of the problem remained unknown until very recently when a sequencing attempt was carried out using MS to look again at the P1a P1b problem using such an approach. To our surprise, we identified two proteins with a similar amino acid composition but divergent sequence (Figs. 2E P1a, P1b) in which the N-terminal amino acid corresponds to pyroglutamine, an amino acid found in *A. mellifera* propolis (Eroglu et al. 2016) and a glutamic/glutamine PTM that blocks Edman degradation (Chelius et al. 2006). Interestingly, both of them appear to have an isoform in which arginine at position 19 is replaced by a histidine. From the higher molecular masses of the nontruncated versions 6642.05 Da (R variant) and 6623.02 Da (H variant) and 6428.93 Da and 6409 Da for the respective truncated forms, is tentative to assume that the forms with larger molecular mass correspond to P1a with the smaller ones corresponding to P1b. Hence, the P1a P1b SNBP components appear to be the products of post-translational cleavage. The gene(s) encoding them are shown in the supplementary materials.

**Fig. 3.**
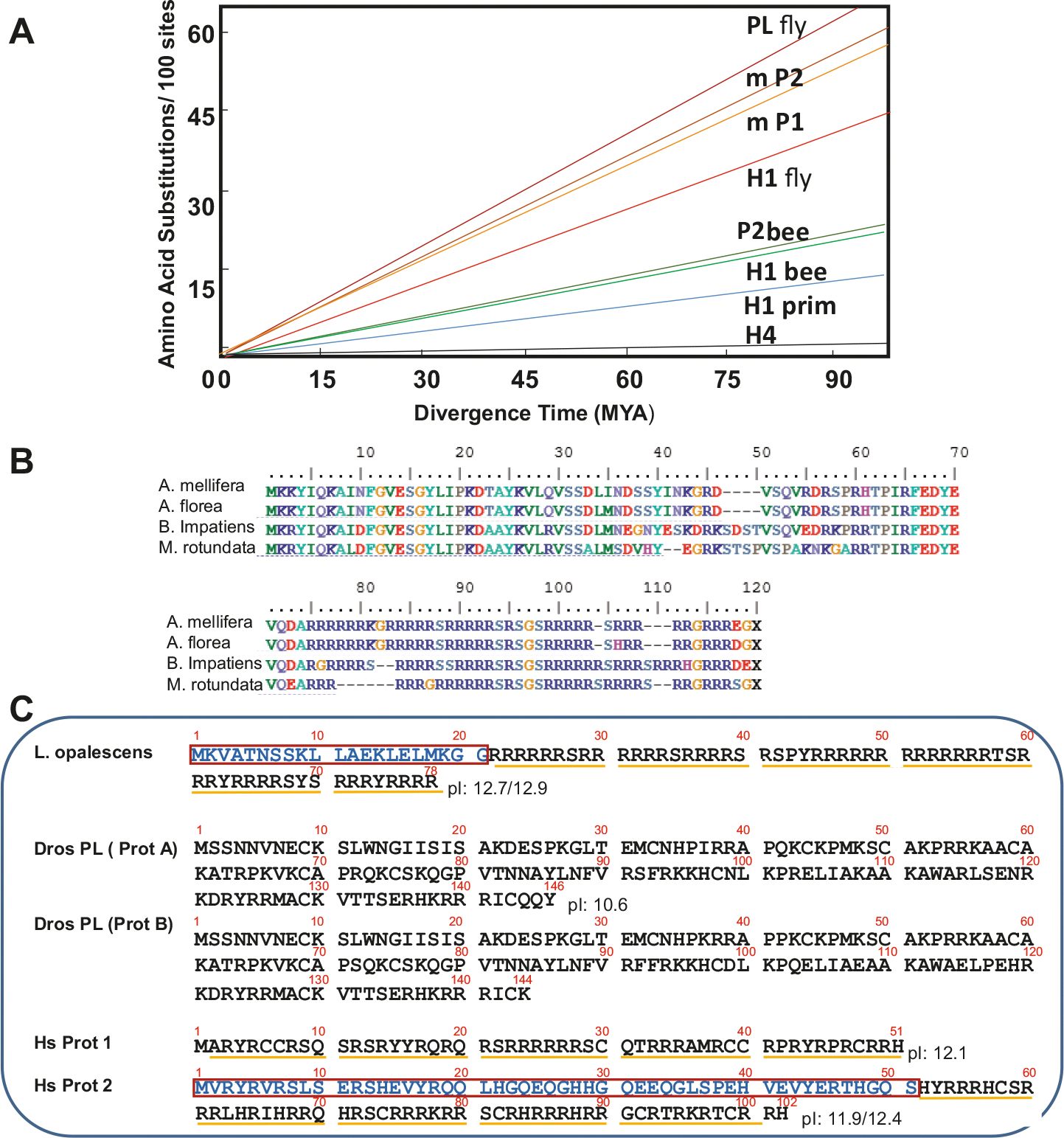
Bee protamine 2 evolution. (A) Evolutionary rates (aminoacidchangeper100 sites) of bee protamine P2 and bee somatic histone H1 compared to *Drosophila* PL (protamines) (Alvi et al. 2013) and primate pP1/pP2 protamines (Retief et al. 1993) and to histones (Isenberg 1978). (B) Protamine sequences and accession numbers for *Apis florea* (red dwarf honey bee) (Aflorea_gi|380025480|ref|XM_003696454.1|:1267–1599 predicted: uncharacterized LOC100871426, mRNA); *Bombus impatiens* (bumble bee) Bimpatiens PROTAMINEnt_(gnl|Bimp_2.0|scf_0083 NT_176502.1); and *Megachile rotundata* (Alfalfa Leafcutter bee) (Mrotundata_gi|383852691|ref|XM_003701811.1|:1684–2010 PREDICTED: uncharacterized LOC100880031, mRNA). (C) Representative protamine sequences from invertebrate and vertebrate organisms. *Loligo opalescence* (opalescent squid) (Lewis et al. 2004); *Drosophila melanogaster* protamines A and B (Jayaramaiah Raja and Renkawitz-Pohl 2005); *Homo sapiens* protamines P1 and P2. The boxed parts of the sequences correspond to protein regions (in blue) of the precursors that become processed by cleavage during spermiogenesis. The underlined regions in yellow indicate the arginine/cysteine clusters characteristic of protamines.

In 2006 when the *A. mellifera* genome sequence became available (Weinstock and Consortium 2006), we initiated an extensive blast analysis using the amino acid sequence obtained from P2. However, because of the short arginine-rich repetitive sequence of P2 (Fig. 2E), a problem to which we have eluded earlier in the introduction, such an effort proved initially unsuccessful. It was not until we directly contacted the Honey Bee Genome Sequencing Consortium that, using a blast search (with the repeat filters turned off) against all protein predictions at BeeBase, they were able to produce the protein sequence shown in (Fig. 2D), which encompasses the P2 precursor sequence. The identification of the gene encoding P2 (supplementary materials) turned out to be very beneficial. Such identification allowed us to identify the related SNBPs from other bees for which genome information is available (Fig. 3) from the Hymenoptera Genome Database. This made it possible to use this information to look at the rate of evolution of the hymenopteran protamines from the superfamily Apoidea of the clade Anthophila (Peters et al. 2017) to which the species used in the analysis (Fig. 3B) belong. When their evolution was compared to that of the primate protamines P1 and P2, and more importantly to the rapidly evolving *Drosophila* PL (see Fig. 3C for representative sequences), their evolution rate was found to be substantially lower, albeit much higher than that of histones (Isenberg 1978) (Fig. 3A).

Regardless of all the difficulties encountered in their identification, the SNBP P1 and P2 from *A. mellifera* are very similar both in size and composition to the arginine-rich protamines of other invertebrate and vertebrate organisms (Kasinsky et al. 2012), and they are likely the products of extensive protein precursor processing. It is this processing, that might ultimately be responsible for the complex chromatin organization transitions observed during spermiogenesis (Fig. 2F) which are characteristic of insects (Kasinsky et al. 2021). The less charged disordered regions of the protein amino acids 1–69 of P2 (Fig. 2D) are likely involved in phase separation transitions (Fig. 2F II–IV), leading in some instances to nucleation and/or a highly compacted state observed once only the highly charged C-terminal portions of the molecules are left (Fig. 2F V).

### Nasonia and the occurrence of cysteine in insect SNBPs

Whereas the honey bee SNBPs could be easily extracted with 0.6 N HCl, the first early attempts to extract these proteins from the testes of *N. vitripennis*, using this procedure, proved to be completely unsatisfactory; hinting at the presence of cysteine. Cysteine is an amino acid frequently observed in other invertebrate and vertebrate organisms (Lewis et al. 2003). The presence of this amino acid in protamines enhances chromatin compaction through the formation of both inter- and intra-protamine disulfide bridges (Balhorn et al. 1991, 1992;Ward and Coffey 1991; Balhorn 2007). When this occurs, it is imperative to alkylate the cysteines before proceeding with the acid extraction. While this is usually feasible with vertebrate organisms where a large amount of sperm material is often available (Soler-Ventura et al. 2018), it adds an important extra layer of complexity in insects, especially in species such as *Nasonia,* where the amounts of sperm (testes) are quite limiting.

**Fig. 4.**
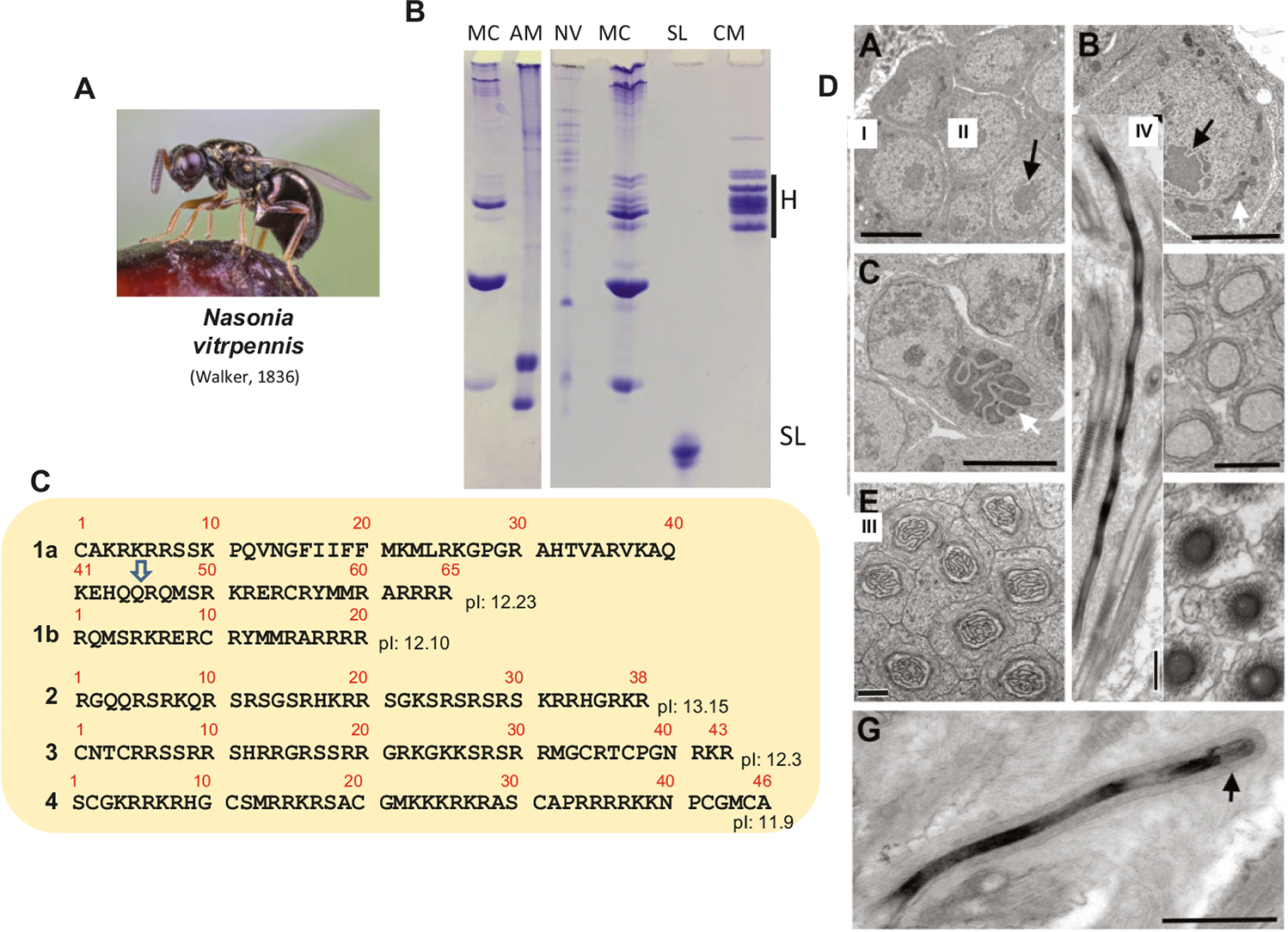
Protamines of the jewel wasp. (A) The jewel wasp *Nasonia vitripennis* (picture provided by Hans Smid). (B) AU-PAGE analysis of the SNBPs of *N.* vitripennis (NV) in comparison to the sperm nuclear basic proteins (SNBPs) of the mussel *M. californianus* (MC), salmon protamine (SL), and chicken erythrocyte histones (CM). (C) Mass sectrometry identified SNBPS from a mature gonad protein extract as shown in (B) NV. (D) Electron micrographs at different stages of spermiogenesis. I and II = longitudinal nuclear sections and III transversal cross section of nuclei of late spermatids. IV is a longitudinal section of mature sperm. Scale bars = 500 nm.

Figure 4B shows the SNBP composition of *N. vitripennis* (NV) in comparison to the *A. mellifera* (AM) SNBPs when the proteins were HCl extracted after pyridylethylation of cysteine (see Materials and methods). The small amounts of material thus obtained with this procedure were directly analyzed by LCMS without any attempts of further fractionation. The results thus obtained are shown in Fig. 4C. Although the approach taken precludes the assignment of any of the respective sequences to bands in the gel (Fig. 4B), none of the results obtained appear to have much resemblance to the P1/P2 bee protamines (Fig. 2E). This is not surprising as *Nasonia* belongs to the hymenopteran family Pteromalidae within the superfamily Chalcidoidea of parasitoid jewel wasps, an early monophyletic group evolutionarily distant from the superfamily Apoidea (Peters et al. 2017). In fact, the chromatin condensation pattern induced by the *Nasonia* SNBPs (Fig. 4D) is distinctively different from that observed for *A. mellifera* (Fig. 2F), with the late spermatids exhibiting a lamellar pattern (Fig. 4D I–III) (Ferree et al. 2019) which is reminiscent of the liquid–liquid phase separation (LLPS) driven chromatin condensation observed in primitive insects (Kasinsky et al. 2021).

### Extensive protein processing in the bush cricket and the cicadas

**Fig. 5.**
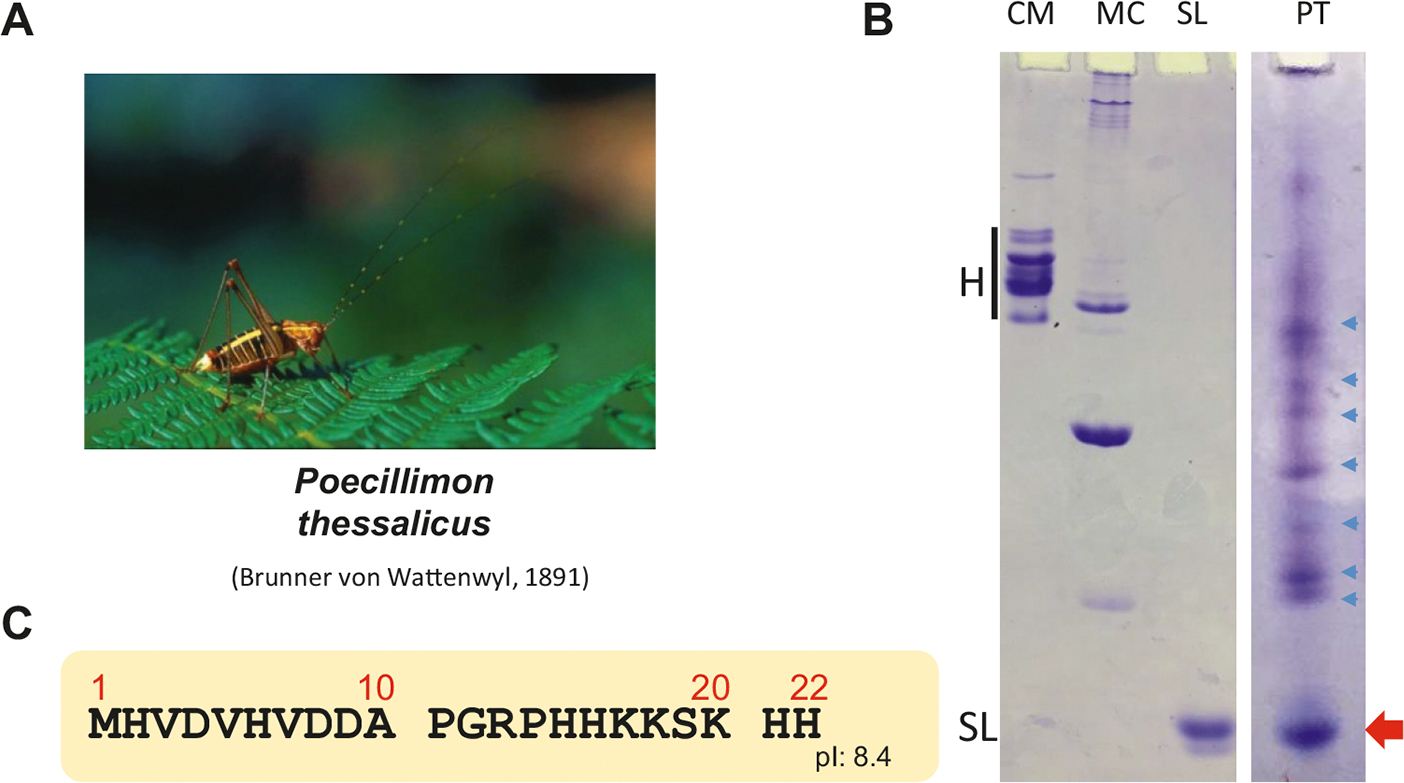
Sperm nuclear basic proteins (SNBPs) of the bush cricket *Poecillimon thessalicus* (A). (B) AU_PAGE comparative analysis of *P. thessalicus* SNBPs. (CM, MC, and SL as in the legend of previous figures). The blue arrows point to potential SNBP precursor proteins. (C) Amino acid sequence determined by N-terminal Edman sequencing of the Reversed phase HPLC purified protein shown by the red arrow in (B).

**Fig. 6.**
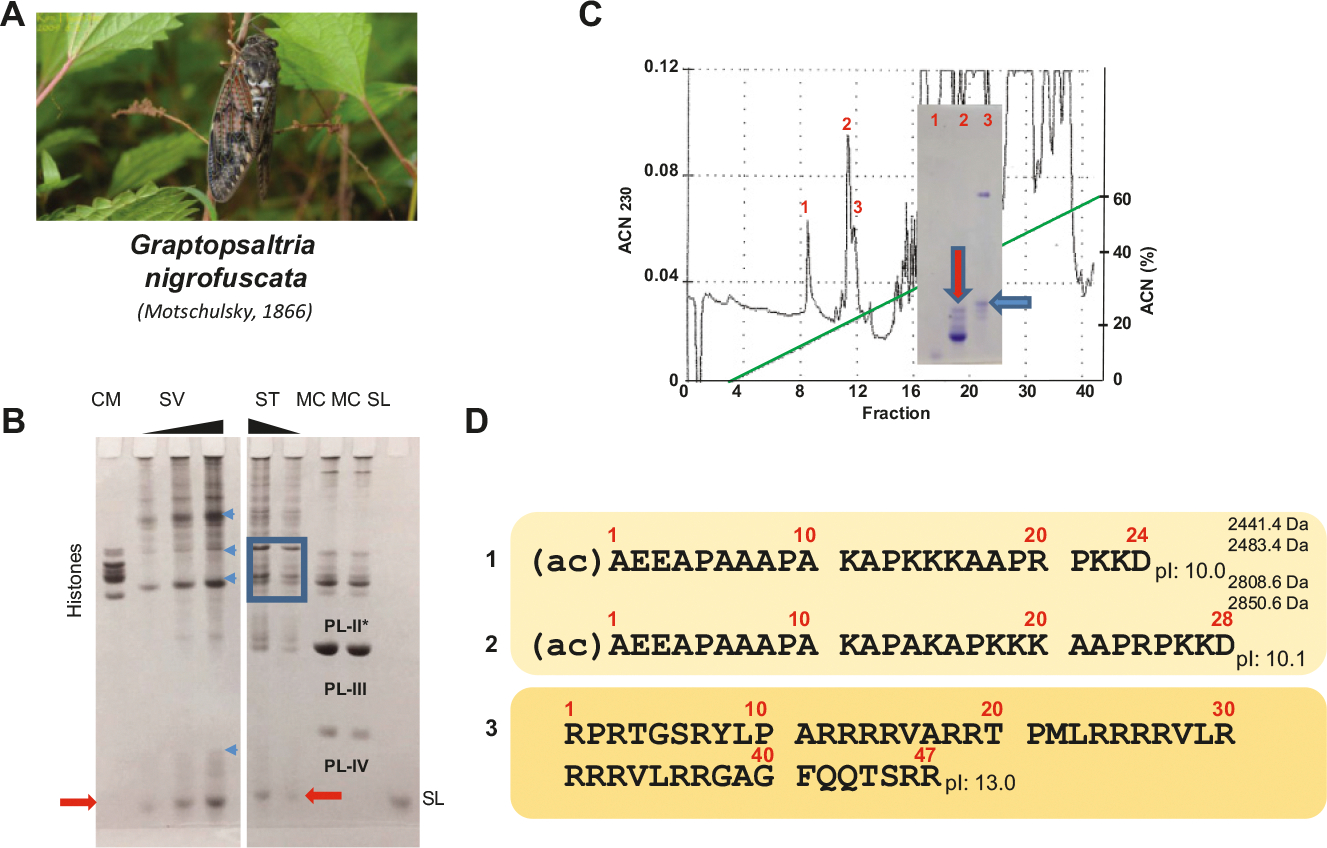
Partial analysis of the sperm nuclear basic proteins (SNBPs) of the large brown cicada *Graptosaltria nigrofuscata* (A). (C) Reversed phase HPLC chromatogram of the sample (SV) shown in (B). The inset shows an electrophoretic analysis of elution peaks 1–3. (B) AU-PAGE of HCl extracts from seminal vesicles (SV) and from spermatheca (ST) in comparison to chicken erythrocyte histones (CM), mussel (MC), and salmine (SL). The blue square indicates the histones from the spermathecal tissue and the blue arrows point to potential SNBP precursor proteins. (D) The fraction indicated by the red arrow in (C) was analyzed by mass spectrometry and the SNBPs identified are shown in (D). The proteins in 1 and 2 were both present in their acetylated (ac) and not acetylated form. The arginine-rich protein in 3 might correspond to the band highlighted by a blue arrow in (C).

Figures 5B and 6B show the SNBP composition of the proteins extracted from the spermatophores of the bush cricket *P. thessalicus* and from the seminal vesicles and spermatheca of the cicada *G. nigrofuscata.* A common feature of the SNBPs of the two species is the presence in both of a prominent electrophoretic band that runs at the same level as the protamine of salmon (salmine = SL). The proteins indicated by the red arrows in Figs. 5B–6C were in both instances purified using rp-HPLC, and their sequences (Figs. 5C and 6D) were analyzed by Edman N-terminal degradation and MS, respectively. Of note, the respective isoelectric points (pIs) of these proteins are low relative to canonical protamines, such as for instance, the bee protamine (Fig. 2E). In Fig. 6D–3, a minor protein with a high pI was identified which does not appear to correspond to any of the bands in Fig. 6C, lane 2. However, because the HPLC fraction corresponding to lane 2 was analyzed by LCMS several years after its purification and considering the high sensitivity of the LCMS approach used in this paper, it is possible that the sequence in Fig. 6D–3 came from cross contamination of lane 2 by lane 3 in Fig. 6C. Unfortunately, the sample from lane 3 was not any longer available at the time of this most recent analysis to confirm this. Nevertheless, the band indicated by a blue arrow in Fig. 6C lane 3 has an approximate molecular mass that would be consistent with that of the arginine rich protein in Fig. 6D–3).

It is interesting that in both in *P. thessalicus* and in *G. nigrofuscata,* other proteins with larger masses were observed (see blue arrows in Fig. 5B PT and Fig. 6B SV). However, as shown in Fig. 6B ST, only the smaller fraction is present in the cicada spermatheca, suggesting that this is the protamine present in mature sperm and that both in the cicada and bush cricket, SNBPs undergo extensive protein cleavage during maturation. Moreover, *G. nigrofuscata* produces two types of sperm that differ in both nuclear volume of the early spermatids and in the length of mature sperm. During copulation, both forms are transferred from the vesicula seminalis to the bursa copulatrix of the female. Fertile sperm accumulate in the spermatheca, where only long sperm survive for any length of time and is used for fertilization (Kubo-Irie et al. 2003).

It is possible that such an intricate processing observed in the bush cricket and cicadas is involved in the protein transition states that, as mentioned earlier, probably lead to the LLPS chromatin condensation that has been observed in other insects of the class Orthoptera and Hemiptera (see fig. 8 in Kasinsky et al. (2021)).

### Insect protamine sequence microheterogeneity

Chromosomal proteins can, in several instances, exhibit amino acid sequence microheterogeneity. For instance, vertebrate linker histones (histones of the H1 family) (Cole 1987) and alligator protamines (Hunt et al. 1996) provide two distinctive examples of such occurrence. The fishfly *Parachauliodes continentalis,* males produce sperm in bundles that swim in cooperation using a synchronous flagellate motion in viscous seminal fluids until they reach the spermatheca (Hayashi 1998).

**Fig. 7.**
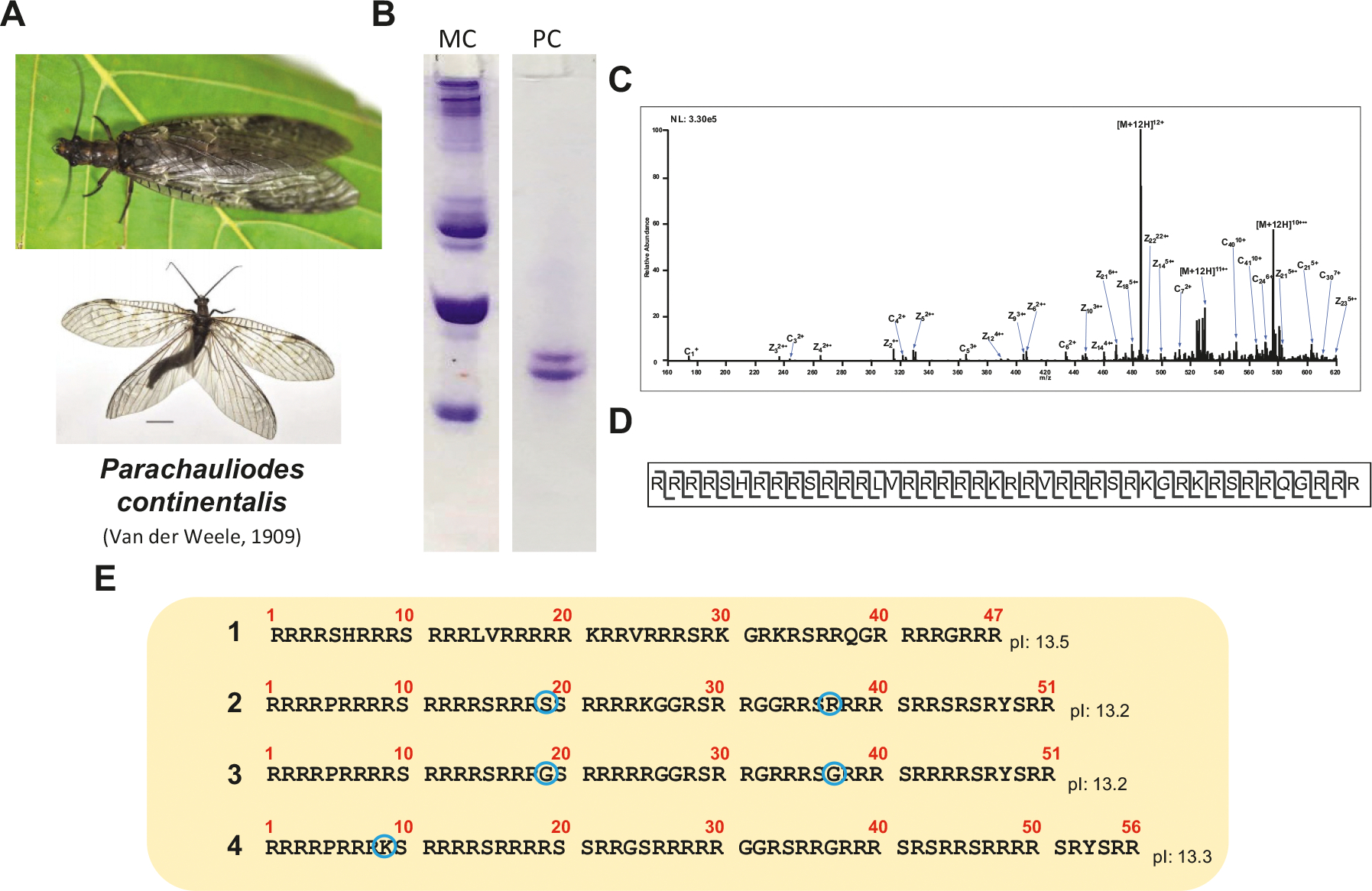
Characterization of the sperm nuclear basic proteins (SNBPs) of the fishfly *Paracauliodes continentalis* (A). (B) Electrophoretic (AU-PAGE) analysis of the HCl-extracted proteins from seminal vesicles (PC) Compared to M. californianus SNBPs (MC). (C and D) A representative depiction of the LCMS approach used for the characterization of *P. continentalis* SNBPs. (C) Precursor ions at 485.56 m/z (*z* = 12) were fragmented by electron transfer dissociation (ETD) to produce the MS2 and selected abundant fragment ions are labeled. (D) Sequence coverage of 485.56 m/z (*z* = 12) *P. continentalis* protamine with molecular mas of 5812.6 Da by ETD. The cleavages depict the c and z^•^ ions observed, giving unambiguous sequence coverage. (E) Table summarizing all the SNBPs detected using the mass spectrometry approach. The blue circles highlight some of the amino acid variations.

Figure 7B PC shows an electrophoretic analysis of the SNBPs extracted from the seminal vesicles of this insect, which basically consists of two prominent bands. The protein sequencing work started initially by conventional Edman degradation of the HPLC-fractionated bands and it has been recently finalized by LCMS (Figs. 7C and 7D). As shown in Fig. 7E, the two bands in Fig. 7B PC consist of a mixture of at least four different proteins with very strong sequence similarity and several amino acid substitutions, suggesting the existence of multiple genes encoding for protamines with sequence microheterogeneity. Their true protamine nature is revealed by their high arginine clusters, as observed in other invertebrate and vertebrate protamines (Lewis et al. 2003; Kasinsky et al. 2012).

### The protamine sequence of Tribolium. When data mining works

**Fig. 8.**
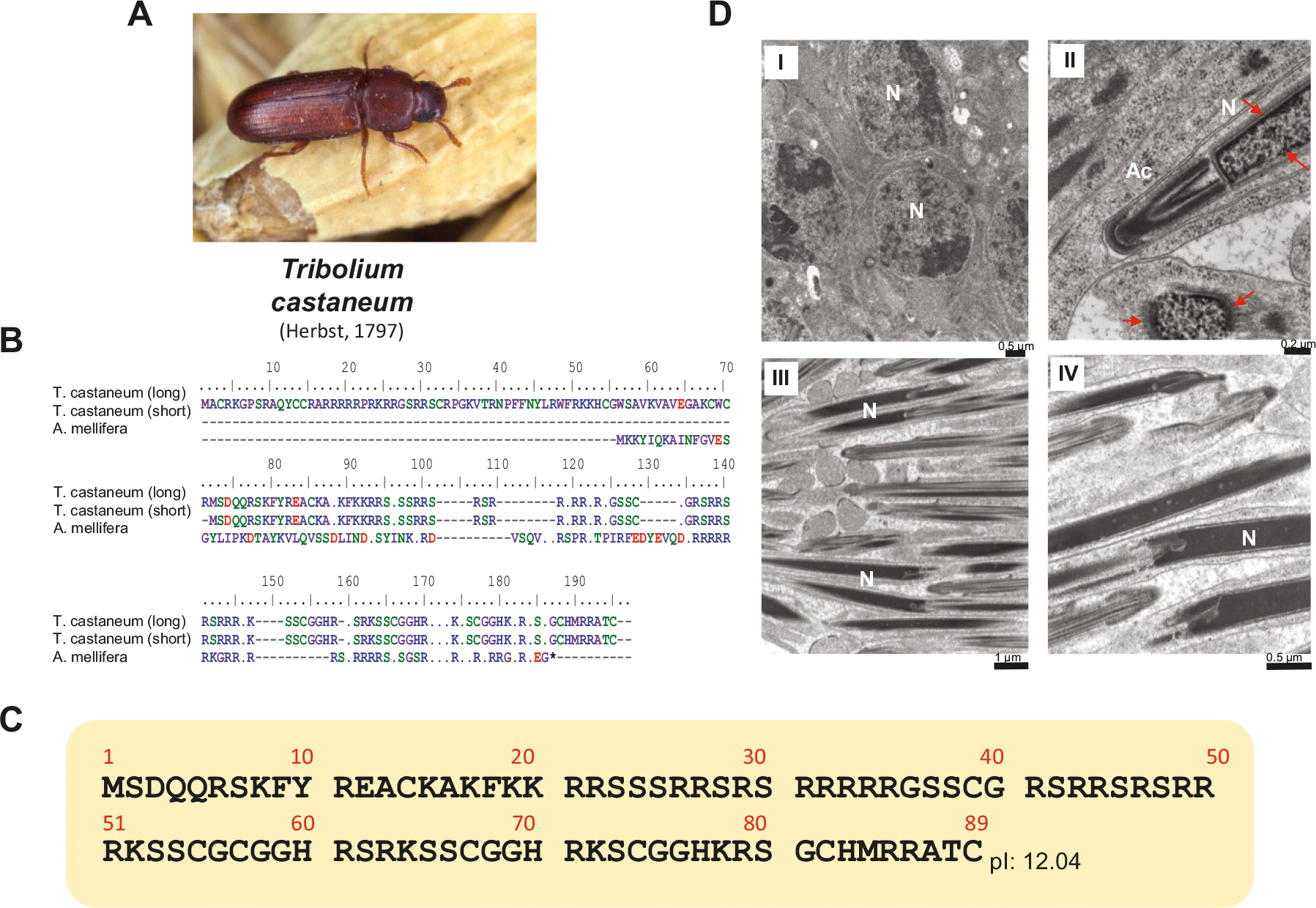
Genome mining identification of the red flour beetle *Trilobium castaneum* (A). (B) Protein identified using *Apis mellifera* sperm nuclear basic proteins (SNBP) (Fig. 2E) in recurrent BLAST searches of the (Tribolium Genome Sequencing et al. 2008) genome on GenBank (project accession AAJJ00000000.2). (C) *T. castaneum* (short) SNBP. (D) Electron microscopy of *Tribolium corretto* showing: I, early spermatids; II, details of the nucleus (N) and acrosome organization (Ac); II, Mature sperm nuclei (N) and IV, an enlarged magnification of the sperm nuclei (N).

Unfortunately, the high interspecific SNBP sequence variability of insects makes it very difficult to use protein sequences from a particular species to gain additional information on other insects for which genomic data are already available. However, the identification of the gene sequence from the *Apis* P2 protamine made it possible to interrogate several insect genomes for for which genomic information is available. In doing so, we were able to retrieve a few candidate gene sequences; in particular, the SNBP sequence for the red flour beetle *T*. *castaneum* was obtained with high confidence. Figure 8B shows the protein alignment between *A. mellifera* and *T. castaneum* based on a BLAST search using gene sequence alignment.

As in the *Apis* P2 protamine gene, the *Tribolium* protamine (Fig. 8C) is encoded by a gene that produces a couple of precursors: a long (177 amino acid) and a short (89 amino acid) (Fig. 8C) peptide both of which very likely undergo protein trimming during spermiogenesis, such as in the case of *A. mellifera* (Fig. 2D). Whether this is the unique protamine for this insect or a few more isoforms are present, like in the case of the *Apis* P1a and P1b protamines, remains to be elucidated.

In contrast to *Nasonia* (Fig. 4D) which exhibits LLPS-driven chromatin condensation during spermiogenesis, the process of sperm chromatin condensation of *Tribolium* (Fig. 8D) is more similar to that of the *Apis* (Fig. 2F), where a contorted chromatin organization, often starting from the nuclear periphery (Fig. 8D II, arrows, and (Dias et al. 2015)), progressively condenses chromatin in the mature sperm.

## Discussion

Figure 3C provides representative sequences for an invertebrate protamine of the squid *L. opalescens*, two fruit fly protamines from *D. melanogaster* and the human protamines P1 and P2. As shown, protein processing by cleavage of the N-terminal domains of their protamine precursors during spermiogenesis is a common occurrence in both vertebrate and invertebrate protamines (Kasinsky et al. 2012). They are additionally characterized by the presence of arginine clusters that may also contain cysteine in their amino acid sequences, as for instance in the cuttlefish (*Eledone cirrhosa*) (Giménez-Bonafé et al. 2002) and in eutherian mammals (Queralt et al. 1995), and they have high isoelectric points of ≥12. They can vary in size, from 30 to 32 amino acids in salmon (see SL (salmine) in PAGE Figures of the paper) to 106 amino acids in the gastropod mollusk *Monodonta turbinata* (Daban et al. 1995). In contrast, the only two genuine SNBP protamine-like proteins from an insect known to date, *Drosophila* Prot A and Prot B, exhibit a much more heterogeneous amino acid composition. Like in other invertebrate PLs (Ausió 1999; Eirin-Lopez and Ausio 2009; Kasinsky et al. 2012), they consist of the basic lysine and histidine in addition to arginine to maintain a basic isoelectric point.

Considering how the number of insect species largely outnumber the species of the rest of the animal kingdom, the finding of additional structural SNBP variability should not be surprising. However, in the species of the Superorder Holometabola studied in this paper (Fig. 1), for the most part their SNBPs correspond to the group of arginine-rich canonical protamines. By contrast, the bush cricket (*P. thessalicus*) (Fig. 5) (Cohort Polyneoptera) and the cicada (*G. nigrofuscata*) (Fig. 6) (Superorder Acercaria), with the exception of the protein shown in Fig. 6D–3), consist of smaller size SNBPs and more divergent amino acid compositions that include lysine and histidine and have a lower pI. Nevertheless, they all share a common feature in that they are expressed as precursor SNBPs that appear to undergo extensive protein editing during spermiogenesis, a feature that is most likely critical for the complex chromatin transitions undergone during the sperm maturation process (Kasinsky et al. 2021). All of this differs significantly from the *D. melanogaster* Prot A and Prot B (Fig. 3C), something that can likely be attributed to their very rapid evolution of the sex related genes in this organism (Haerty et al. 2007), as in the primate protamine lineage (Fig. 3A). However, unlike in mammals, there is a gain and loss of genes encoding *Drosophila* SNBPs that appears to be driven by driven by genetic conflicts between sex chromosomes (Chang et al. 2023) and their encoded protamines impart an epigenetic identity of paternal chromosomes, a characteristic that might be shared with other insects (Dubruille et al. 2023). The lowest evolutionary rate of the P2 in Apoidea might account for the more conserved compositional amino acid variability or of their protamine genes, something that might be more definitely established once the gene(s) for P1 in *A. mellifera* are identified.

The presence of pyroglutamic acid, a PTM that substantially extends the half-life of proteins (Huang et al. 2008), in bee protamines is of interest. Such a modification is part of the P1 protamine precursor processing and is likely imparted by glutaminyl ciclases (Huang et al. 2008), such as those found in mammals and insects (Koch et al. 2012; Adamson et al. 2013; Wu et al. 2017), upon post-translational cleavage of the yet to be identified P1 precursor. Such PTM could potentially be related to the fertilization biology of this insect in which the queen bee stores the sperm in the spermatheca for very long periods of time (Baer et al. 2016).

In closing, we present our results on a limited number of species analyzed during the last 35 years. We analyzed more species than the ones presented here but we could not get conclusive data for several of them due, amongst other things, to the limiting amounts of material available for their proper analysis. As the number of insect genomes has been continuously dramatically increasing in recent years, one would expect that SNBP identification for many of these organisms would become easier. Yet, trying to define a protamine based on high arginine content is not an easy task. Many ribosomal proteins are also arginine rich, and the annotation for SNBP genes in these genomes is almost nonexistent as it is usually compromised by the heterogeneous nonarginine component of the N- and C-terminal regions of the precursor protamines and/or the amino acid variability itself of the protamine trying to be identified.

With each completely unexpected challenging twist of the protein results described here, of which the *Apis* story provides a good example, it felt at times that studying the insect SNBPs was like opening a Pandora’s Box of fascination and difficulty. The preliminary work started here underscores the complexity and variability of the SNBP composition in this group of invertebrates. Incomplete as the information provided by our paper might be, from the *Apis* protamines to the *Tribolium* SNBP, it provides a glimpse of the enormous complexity of insect SNBPs. We hope it will spearhead a much needed interest in this topic.

## Supplementary Material

Ausio-BiochemCellBiol-2024 supp

Supplementary data are available with the article at https://doi.org/10.1139/bcb-2023-0363.

## Data Availability

All portions of the data underpinning the work are fully available.
