# Supplementary material for: Protamines and the sperm nuclear basic proteins Pandora’s Box of insects
Source: Biochem Cell Biol. Author manuscript; Available in PMC 2025 Jul 2. (PMC12216123; doi:10.1139/bcb-2023-0363)
Supplement: Ausio-BiochemCellBiol-2024 supp [file NIHMS2089843-supplement-Ausio-BiochemCellBiol-2024_supp.docx]

**Supplementary materials**

Blast analyses of *A. mellifera* P1a/b and P2 protamine genes performed by Kim C. Worley and Ryszard Maleszka of the Human Genome Sequencing Center at the Department of Molecular and Human Genetics, Baylor College of Medicine on February 2010, using an early *Apis mellifera* genome (left) and on a recently updated genome (right).
